# Terrestrial vegetation redistribution and carbon balance under climate change

**DOI:** 10.1186/1750-0680-1-6

**Published:** 2006-07-25

**Authors:** Wolfgang Lucht, Sibyll Schaphoff, Tim Erbrecht, Ursula Heyder, Wolfgang Cramer

**Affiliations:** 1 Potsdam Institute for Climate Impact Research, PO Box 6012303, D-14412 Potsdam, Germany; 2 Institute of Geoecology, Potsdam University, PO Box 601553, D-14415 Potsdam, Germany

## Abstract

**Background:**

Dynamic Global Vegetation Models (DGVMs) compute the terrestrial carbon balance as well as the transient spatial distribution of vegetation. We study two scenarios of moderate and strong climate change (2.9 K and 5.3 K temperature increase over present) to investigate the spatial redistribution of major vegetation types and their carbon balance in the year 2100.

**Results:**

The world's land vegetation will be more deciduous than at present, and contain about 125 billion tons of additional carbon. While a recession of the boreal forest is simulated in some areas, along with a general expansion to the north, we do not observe a reported collapse of the central Amazonian rain forest. Rather, a decrease of biomass and a change of vegetation type occurs in its northeastern part. The ability of the terrestrial biosphere to sequester carbon from the atmosphere declines strongly in the second half of the 21^st ^century.

**Conclusion:**

Climate change will cause widespread shifts in the distribution of major vegetation functional types on all continents by the year 2100.

## Background

The distribution of the world's vegetation has changed with past changes in climate and will continue to do so in the future. Due to rapidly increasing greenhouse gas concentrations, climate changes now more quickly than it has been doing for a long time [1] – but the pattern is irregular due to the complex changes in weather patterns, warming and rainfall change. So how much will vegetation change and where will it change most dramatically? Being able to answer these questions, even roughly, is important for two reasons. First, much of human well-being depends on ecosystems, due to the many services they provide [2]. Second, land ecosystems contain large amounts of carbon which could be released as a consequence of major changes – they therefore may accelerate or slow down climate change substantially [3-5] (accelerate, e.g. due to increasing carbon emissions from organic soils, wildfires or forest die-back, or slow down, e.g. through increased vegetation growth and storage in dry or cold soils).

Terrestrial vegetation responds to climate change on several levels. Changes in temperature, precipitation, light and nutrient availability, and in atmospheric CO_2 _concentration influence plant biochemistry and physiology as well as the allocation of carbon to long- or short-lived plant parts such as leaves, stems and roots. Additionally, plants have evolved different functional strategies to cope with adverse conditions such as drought, cold or inundation (for example, the evergreen and the deciduous strategies of trees), therefore changes in these conditions eventually lead to changes in the species composition of an ecosystem – even if several decades may be needed for the process.

Mapping the outcome of the complex interplay of these processes has become possible due to the development of Dynamic Global Vegetation Models (DGVMs) [6], which simulate the terrestrial balances of carbon and water as well as the temporal development of vegetation in response to changing climate. The geographical pattern of vegetation emerges as a result of different responses of plant functional types to climate, with respect to productivity, bioclimatic constraints, access to resources and space, and sensitivity to natural disturbances such as fire.

Presently, the land biosphere is a net sink of carbon [7]. Most simulations of the land biosphere's response to future climate change (as simulated by climate models) show a decline in this sink beginning around the middle of the 21^st ^century [4,8-10], with some scenarios even showing a net carbon loss by the end of the century [3,9,11]. The magnitude of this terrestrial feedback on climate is projected to be an additional increase in atmospheric CO_2 _concentration of between 20 and 200 ppm, implying an additional increase in temperature of between 0.1 K and 1.5 K [12].

Looking beyond these global numbers, all these simulations contain dramatic regional changes in vegetation structure and composition, in some cases of catastrophic extent. In some model simulations, for example, a collapse of parts of the Amazonian rain forest occurs by the year 2100 due to strongly decreasing rainfall [13]. A decline in boreal forest area due to increasing heat stress on boreal trees has been reported [11]. Another example is a transition from temperate savannah to subtropical woodland for a highland location in Africa [9]. Changes such as these would imply a significant change in the composition and structure of the respective ecosystems – however, they differ depending on the greenhouse gas emission scenario, climate scenario and biosphere simulation model used.

As a step towards the identification of a more robust assessment, we use the state-of-the-art LPJ-DGVM [14,15] and present results for change from the present in natural vegetation for two scenarios of climate change, selected to represent a wide range of potential futures from moderate (though not weak) to strong change by 2100. The consequences of moderate climate change (temperature increase over land: 2.9 K) were computed for ECHAM5 climate model projections under the SRES-B1 emission scenario (rising to 550 ppm CO_2 _in 2100). The consequences of strong climate change (temperature increase over land: 5.3 K) were computed for HadCM3 climate model projections under the SRES-A2 emission scenario (rising to 856 ppm CO_2 _in 2100).

## Results

### Changes of vegetation patterns

**Figure 1:**
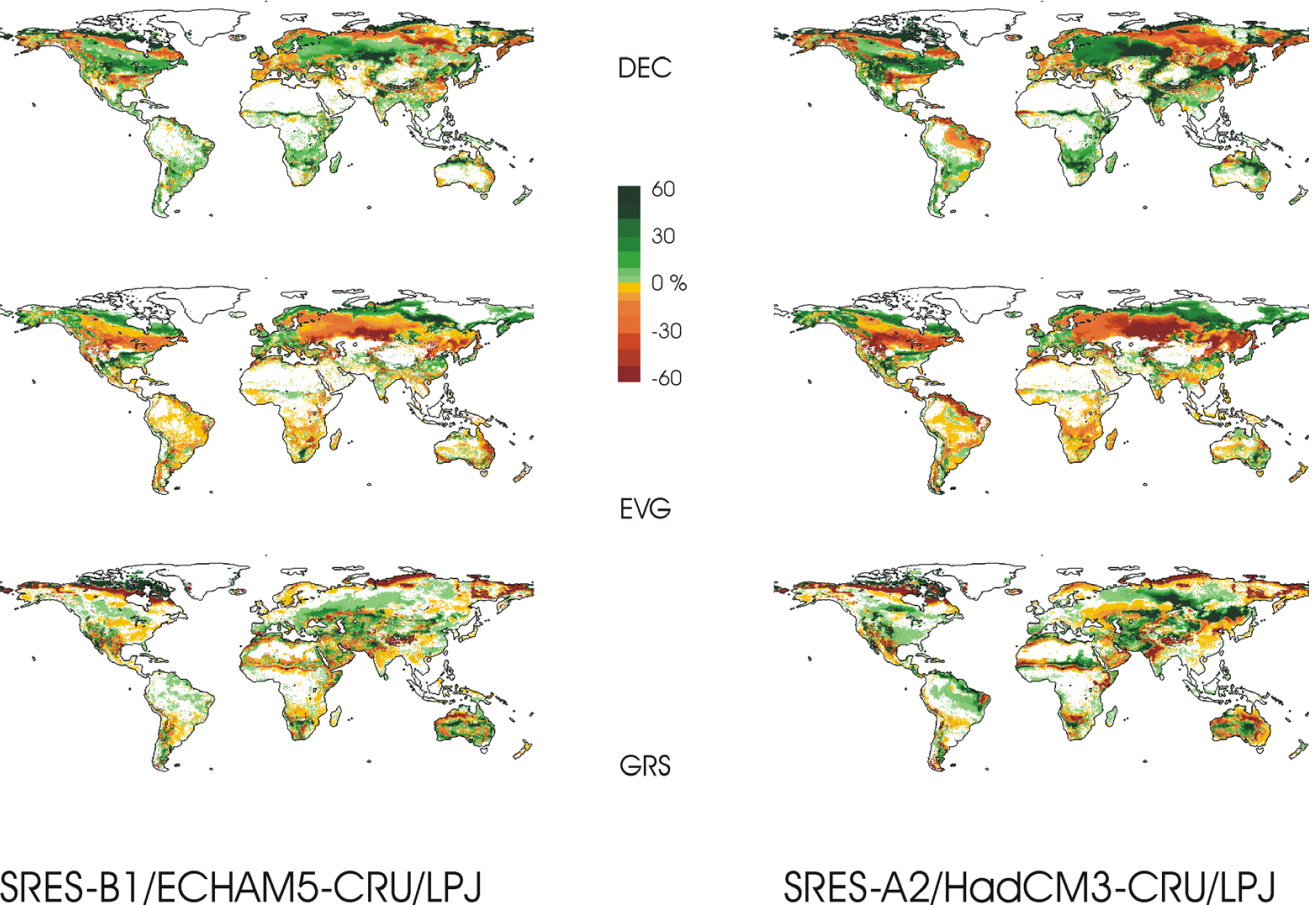
**Changes in global vegetation distribution between 2100 and 2000**. Simulated changes 2100–2000 of the fractional cover of deciduous woody (top), evergreen woody (middle) and non-woody (bottom) vegetation functional types for a moderate (SRES-B1, normalised to 1961–90 observed CRU dataset means, Echam5 climate change simulations) and a strong climate change scenario (SRES-A2, normalised to 1961–90 observed CRU dataset means, HadCM3 climate change simulations). Simulations with the LPJ-DGVM.

Figure 1 shows simulated changes in the cover fraction of major vegetation types. Patterns of change are generally similar for moderate and for strong climate change but are more pronounced and extensive for stronger warming and atmospheric CO_2 _concentration.

Arctic tundra disappears in northern Eurasia, where warming is disproportionately strong, as deciduous woodlands expand. In northern Canada, new tundra is formed on the Arctic fringe that at present is only sparsely or not vegetated.

Boreal forests are affected by a number of large-scale changes. Evergreen vegetation increases its presence along the northern edge of the boreal zone. Further to the south, although in central western Eurasia and in Canada forests remain largely mixed, a widespread shift toward more deciduousness occurs. At the southern edge of the boreal forest, where it borders the steppe in central Asia and Canada, a recession of forest cover is observed in several areas due to increasing drought. In the stronger climate change scenario, the boreal forest collapses or changes into an open woodland in southern eastern Siberia, central western Siberia, and to the southwest of Hudson Bay in Canada due to increased mortality caused by heat stress. One mechanism by which this occurs is peak-summer heat stress on boreal species in areas where continentally cold winters prevent an invasion of temperate species.

In the temperate zone, evergreen plant functional types increase their fractions at the expense of deciduous vegetation in several regions, for example in the southeastern US, in Europe and in parts of eastern China. The savannahs and woodlands of South America and in southern Africa, on the other hand, show increased deciduousness.

The vegetation composition of the tropical evergreen zone is largely unaffected by climate change on the level of functional strategies. The simulations do not show a collapse of the Amazonian rain forest even for strong climate change. A decline in forest area is observed, however, along its eastern fringe.

In several regions with savannah, for example in southern Africa, grasslands experience woody encroachment. In many semi-arid regions, increased water use efficiency due to higher atmospheric CO_2 _concentrations leads to an increase in the herbaceous or grassy cover. Grass cover also increases where forests recede. Regional changes in precipitation, however, may lead to a net decline of non-woody vegetation cover in the affected regions. Increases and decreases of grass cover show a strongly differentiated spatial pattern around the globe.

### Changes in terrestrial carbon storage and exchange

In these simulations, the biosphere contains 228 and 205 GtC (1 GtC = 1 billion tons of carbon) more carbon in the year 2100 than it did in the year 2000, for the moderate and the strong scenario, respectively. Vegetation biomass increases by 131 and 125 GtC, and the sum of carbon contained in litter and soils by 97 and 80 GtC. Net primary production increases by 18 and 28 GtC/yr, soil respiration by 17 and 24 GtC. Emissions from fires increase by 3.3 and 5.3 GtC/yr, respectively. As a consequence, the terrestrial biosphere's carbon sink is 2.4 and 1.6 GtC/yr smaller in 2100 than it is today (current sinks, 1971–2000: 3.0 and 1.9 GtC/yr; future sinks 2071–2100: 0.6 and 0.3 GtC/yr).

**Figure 2:**
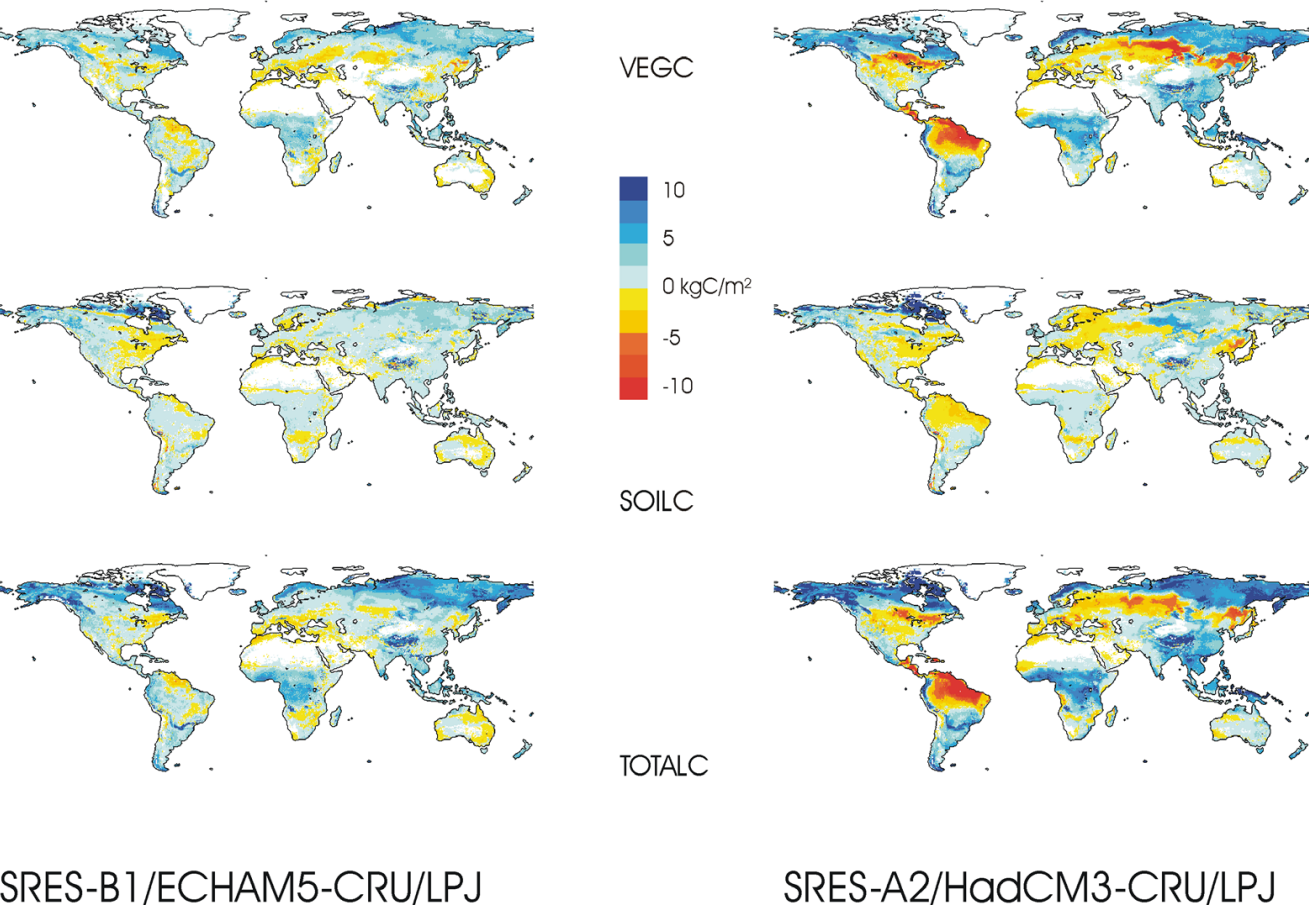
**Changes in global vegetation and soil carbon content between 2100 and 2000**. Simulated changes 2100–2000 in vegetation (top), soil (including litter), and total (bottom) carbon content for the two climate change scenarios (see caption of Fig. 1). Simulations with the LPJ-DGVM.

Figure 2 shows that these numbers are the cumulative result of a differentiated spatial pattern that is more pronounced for stronger climate change. The high northern latitudes act as a considerable sink of carbon as warming and carbon fertilisation stimulate vegetation growth. The boreal and temperate mid-latitudes lose carbon as biomass declines regionally. For strong climate warming, heterotrophic soil respiration is additionally stimulated. The African tropical and subtropical zone acts as a sink as carbon fertilisation and increased water use efficiency support more vegetation. In the strong climate change scenario, biomass strongly declines in the eastern reaches of the tropical rainforests of South America, in a region that is considerably larger than the region in which forest type changes substantially.

**Figure 3:**
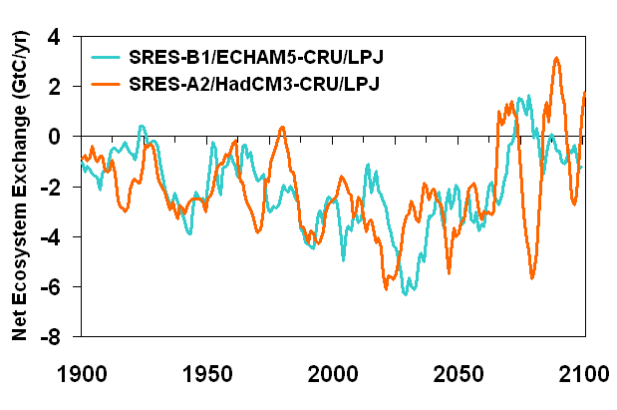
**Temporal evolution of net ecosystem exchange**. Simulated temporal evolution of net land surface carbon exchange (net primary production – soil respiration – fire emissions) for the two climate change scenarios (see caption of Fig. 1). Simulations with the LPJ-DGVM. Negative values denote a carbon sink.

Figure 3 shows the temporal evolution of net carbon exchange between the terrestrial land surface and the atmosphere between 1900 and 2100 for the two scenarios. The terrestrial carbon sink currently achieved will increase until around 2025, then decline. At the end of the century, periods characterised by net terrestrial carbon release become more frequent and the average sink strength drops to close to zero. Major excursions toward the end of the century are related to periods with larger-than-average extremes in simulated climate. Exceptionally dry years with increased wildfire activity (carbon source) are first followed by re-growth during a period with slightly more precipitation (sink), then by renewed fire activity (source).

## Discussion

A major unknown in projecting future vegetation change is the disputed magnitude of carbon fertilisation effects on plant growth. In keeping with short-term experimental data [16,17], DGVMs generally assume a diminishing but persistent stimulation of carbon assimilation with rising atmospheric carbon dioxide concentration and largely invariant allocation of carbon gains to plant compartments. Both of these assumptions remain under dispute in terms of their magnitude, persistence, and general applicability under nutrient and water constraints [18].

A source of uncertainty is also the spatial distribution of future changes in precipitation. Climate models differ considerably in the spatial pattern of their projections, but these changes are a first-order driver of biome structure. Uncertainty also remains due to gaps in generalised knowledge about the extent and mechanisms by which plants and ecosystems are able to adapt to change, and the thresholds beyond which compensatory mechanisms fail. The existence of such thresholds is well-known from the occasionally devastating regional effects on vegetation of years with anomalously extreme climate.

Future changes in nutrient limitations of vegetation growth, particularly with respect to nitrogen and phosphorous, may play an important role in some areas. The LPJ-DGVM assumes leaf nitrogen contents that optimise photosynthesis, but does not simulate allocation shifts away from leaves in response to nitrogen limitations. Simulated biomass increases are, however, not of a magnitude that would likely lead to general nutrient limitation on the global scale, though regional effects are to be expected. Increased water demand of vegetation at higher air temperatures is partly compensated in the LPJ-DGVM simulations by decreases in transpiration under higher ambient CO_2 _concentration. This effect is closely intertwined with concurrent shifts in vegetation abundance and composition [19]. Projections of future fire frequency and severity in LPJ-DGVM simulations depend on the evolution of fuel load, fuel moisture and climatic fire probability, though fire occurrence in ecosystems under environmental stress may be influenced by additional factors.

In our simulations we do not observe a full-scale die-back of the Amazonian rain forest even under strong warming. The pattern of biomass reduction differs from that produced by the vegetation component of the HadCM3 simulation [13], but is similar to a pattern observed with a different DGVM [10]. We suggest two explanations. First, HadCM3's simulated precipitation for the present is lower in some areas than observed. Our normalisation of climate model data to observed averages increases precipitation to observed levels, and the rainforest survives the subsequently simulated reduction, though biomass is lost in the eastern part of the region. Second, we do not compute feedbacks between climate and vegetation, which have been shown to enhance desiccation by 20% [20].

One of the mechanisms by which vegetation biomass is reduced in our simulations of Amazonia is an increase in fire frequency due to reduced precipitation and enhanced litter production. This causes a younger forest age with forest biomass reduced to nearly half of its present value. Where the forest manages to re-establish after fires, the biome type remains unchanged. Forest is replaced by non-woody vegetation in those regions where the frequency and severity of fires prevents its re-establishment. Carbon emissions from fire increase most strongly in northwestern South America, large parts of southern Africa, the Sahel, Australia and India.

The simulated northward expansion of the boreal forest is generally accepted, and there is evidence for a recession of the southern boreal drought-induced tree line [21]. Little substantial discussion exists of the potential causes of boreal forest die-back due to heat stress, possibly due to high peak tissue temperatures. Mortality of stressed boreal trees is increased due to insect infestation and subsequent fire. However, overall vegetation dynamics are complicated by changes in permafrost thawing depth and snow cover, and by potential effects of carbon fertilisation.

## Conclusion

The spatial pattern of the world's vegetation will change under climate change [22-24]. But by how much and where? The answer given by current research has to remain tentative, but all indications are that the changes will be wide-spread if atmospheric CO_2 _increase and warming are not limited to a small magnitude. It would be unwise to treat these changes lightly. Shifts in the spatial distribution of vegetation types signal severe change in the underlying ecosystems, with effects on a large number of species. The modelling, observational and political challenges that follow for biosphere and ecosystem research are as large, if not larger, than those encountered in climate system research. The biosphere will continue to remove anthropogenic carbon from the atmosphere but with diminishing strength in the second half of the 21^st ^century. Large regions of the globe can be identified that are projected to be either long-term sinks or sources of carbon.

Vegetation models of the next generation will be challenged to provide an improved basis for determining the impact of climate change on vegetation distribution, structure and properties. The processes to be modelled, their physiological basis and interlinkages on the level of ecosystems will require review, discussion in terms of relevance and priority, and integration into new model versions. Close links between biosphere modellers, field ecologists, plant physiologists, biogeographers and experimentalists in biogeochemistry will be required for achieving this advance [25].

Climate change is an important but by far not the only pressure on the terrestrial biosphere. Still expanding land use and deforestation as well as chemical pollution, interregional exchange of species and, perhaps, more widespread genetic engineering in the future will in all likelihood rival or even overshadow the effects of climate change. Today's tropical forests, for example, are under pressure mainly from socioeconomic drivers that are unrelated to but will interact with climate change.

The implications of these findings for policy are threefold. First, biome change is an important dimension in which the degree of acceptable climate change has to be evaluated. Second, the saturation and subsequent decline of terrestrial carbon storage is a factor to be taken into account in discussions of mitigation and adaptation policies. Third, a substantial focus on climatic and non-climatic causes of ecosystem change is required in the next several years to advance the reliability of available projections of land biosphere change.

## Methods

### The LPJ-DGVM

The LPJ-DGVM [14,15] is a process-based model of key ecosystem processes that govern terrestrial biogeochemistry and biogeography. A Farquhar-Collatz photosynthesis scheme is coupled to a two-layered soil hydrological scheme for daily simulations of gross primary production and plant respiration, including effects of drought stress on assimilation and evapotranspiration, depending on tissue-specific C:N ratios, biomass and phenology, and using a modified Arrhenius temperature-dependent formulation. Assimilated carbon is allocated annually to four pools (leaves, sapwood, heartwood and fine roots) to satisfy a set of allometric and functional relations. Leaf and root turnover, as well as plant mortality, feed a litter pool, a slow and a fast soil carbon pool that decay depending on soil temperature through a modified Arrhenius formulation, and soil moisture. Vegetation functional differences are represented by seven woody and two herbaceous plant functional types (PFTs) differentiated by their physiological, physiognomic and phenological attributes. These may co-exist at any location, depending on plant competition for resources and space, and a set of environmental constraints. Fire disturbance is simulated as a function of a threshold litter load and surface soil moisture, permafrost was not modelled. A selection of model validation is contained in [14,19,26-28].

### Modelling protocol

Climate model data were interpolated to 0.5 degrees resolution and normalised in each pixel to the observed 1961–90 CRU2002 climatology [29] mean value. For Echam5 [30], the global mean temperature over land remained nearly unchanged as a consequence, while for HadCM3 [31] it was increased by about 1 K. Individual pixels will, however, have experienced different amounts of normalisation. Precipitation was normalised using a relative factor. A spinup of LPJ was performed lasting 900 years using CRU data to place carbon pools into a realistic initial state. This was followed by a second shorter spin-up with normalised GCM data to allow adjustments of vegetation distribution to the characteristics of GCM climate. Transient runs from 1860 to 2100 followed, of which the averages of the periods 1971–2000 and 2071–2100 were evaluated. The two combinations of scenario and model used were selected to represent a moderate (but not weak) and a strong example of potential futures from among the range of results produced by a larger number of combinations of scenarios and models.

## Competing interests

The author(s) declare that they have no competing interests.

## Authors' contributions

WL designed and supervised the analysis and wrote the manuscript. SS carried out the analysis and co-devised the design. TE and UH contributed data handling and analysis, WC analysis, writing and supervision.
